# Down-Regulation of miR-23a-3p Mediates Irradiation-Induced Neuronal Apoptosis

**DOI:** 10.3390/ijms21103695

**Published:** 2020-05-24

**Authors:** Boris Sabirzhanov, Oleg Makarevich, James Barrett, Isabel L. Jackson, Alan I. Faden, Bogdan A. Stoica

**Affiliations:** 1 Center for Shock Trauma Anesthesiology Research, Department of Anesthesiology, University of Maryland School of Medicine, Baltimore, MD 21201, USA; Oleg.Makarevich@som.umaryland.edu (O.M.); james.barrett@som.umaryland.edu (J.B.); afaden@som.umaryland.edu (A.I.F.); 2 Division of Translational Radiation Sciences (DTRS), Department of Radiation Oncology, University of Maryland School of Medicine, Baltimore, MD 21201, USA; ijackson@som.umaryland.edu; 3 VA Maryland Health Care System, Baltimore VA Medical Center, Baltimore, MD 21201, USA

**Keywords:** neuronal apoptosis, radiation, microRNA (miR), Puma, Noxa, Bim, MOMP

## Abstract

Radiation-induced central nervous system toxicity is a significant risk factor for patients receiving cancer radiotherapy. Surprisingly, the mechanisms responsible for the DNA damage-triggered neuronal cell death following irradiation have yet to be deciphered. Using primary cortical neuronal cultures in vitro, we demonstrated that X-ray exposure induces the mitochondrial pathway of intrinsic apoptosis and that miR-23a-3p plays a significant role in the regulation of this process. Primary cortical neurons exposed to irradiation show the activation of DNA-damage response pathways, including the sequential phosphorylation of ATM kinase, histone H2AX, and p53. This is followed by the p53-dependent up-regulation of the pro-apoptotic Bcl2 family molecules, including the BH3-only molecules PUMA, Noxa, and Bim, leading to mitochondrial outer membrane permeabilization (MOMP) and the release of cytochrome c, which activates caspase-dependent apoptosis. miR-23a-3p, a negative regulator of specific pro-apoptotic Bcl-2 family molecules, is rapidly decreased after neuronal irradiation. By increasing the degradation of PUMA and Noxa mRNAs in the RNA-induced silencing complex (RISC), the administration of the miR-23a-3p mimic inhibits the irradiation-induced up-regulation of Noxa and Puma. These changes result in an attenuation of apoptotic processes such as MOMP, the release of cytochrome c and caspases activation, and a reduction in neuronal cell death. The neuroprotective effects of miR-23a-3p administration may not only involve the direct inhibition of pro-apoptotic Bcl-2 molecules downstream of p53 but also include the attenuation of secondary DNA damage upstream of p53. Importantly, we demonstrated that brain irradiation in vivo results in the down-regulation of miR-23a-3p and the elevation of pro-apoptotic Bcl2-family molecules PUMA, Noxa, and Bax, not only broadly in the cortex and hippocampus, except for Bax, which was up-regulated only in the hippocampus but also selectively in isolated neuronal populations from the irradiated brain. Overall, our data suggest that miR-23a-3p down-regulation contributes to irradiation-induced intrinsic pathways of neuronal apoptosis. These regulated pathways of neurodegeneration may be the target of effective neuroprotective strategies using miR-23a-3p mimics to block their development and increase neuronal survival after irradiation.

## 1. Introduction

Radiation therapy is one of the most important interventions targeting central nervous system tumors. Unfortunately, radiotherapy has significant side effects [1], and clinical studies indicate that 30% of long-term brain tumor survivors older than 50 years who receive radiotherapy develop dementia, with an additional 20% showing significantly impaired short-term memory and other functional neurological deficits [2]. Patients that received whole-brain radiotherapy may develop progressive dementia within 5–36 months [3], and an increased risk of brain impairments may also result from exposure to ionizing radiation (IR) during other medical procedures, as well as from nuclear/radiation accidents or cosmic radiation [4,5,6].

Whole-brain irradiation in a mouse glioma model is associated with neurological dysfunction, including memory deficits [7]. Studies have shown that IR induces DNA damage and activates neuronal apoptotic pathways, leading to neuronal loss [8,9,10]. Agents such as valproic acid [11] and lithium [12,13] may down-regulate the expression of pro-apoptotic Bcl-2 genes and attenuate IR-induced neuronal apoptosis, improving cognitive performance. Kukoamine A was also shown to attenuate radiation-induced neuronal cell death by inhibiting caspase-dependent apoptosis [14]. Thus, neuroprotection could be an effective strategy to prevent the development of neurocognitive deficits after brain irradiation. However, this approach would require a much better understanding of the molecular mechanisms involved in neuronal apoptosis after ionizing radiation and their key regulators than is currently available [15].

MicroRNAs (miRs) are short (approximately 20 nucleotides) noncoding RNAs that negatively regulate gene expression at the post-transcriptional level by binding to the 3′-untranslated region (UTR) of target mRNAs, leading to their degradation and/or translational inhibition [8]. One strand of the mature miRNA binds to Argonaute (Ago) proteins to form the RNA-induced silencing complex (RISC), where miRs act as a template for the recognition and cleavage of complementary mRNA. miRs play important roles not only in physiological neuronal activities but may also act as pathophysiological agents in various central nervous system (CNS) disorders [16,17,18], in part through modulating neuronal cell death pathways [19,20].

Two prior studies examined changes in adult brain miRs after low dose (≤1 Gy) X-ray irradiation [21,22]; however, they reported neither neuron-specific information nor the mechanistic role of identified miRs in IR-induced neuronal cell death. Thus, the role of miRs changes as regulators of neuronal apoptosis pathways following irradiation has yet to be examined.

In the present study, we demonstrated that neuronal exposure to IR in vitro and in vivo reduces the levels of miR-23a-3p inducing the activation of specific pro-apoptotic Bcl2 family molecules and intrinsic apoptotic mechanisms. Notably, the administration of a miR-23a-3p mimic inhibits the IR-dependent activation of mitochondrial apoptosis pathways and reduces neuronal cell death.

## 2. Results

### 2.1. Expression of Pro-Apoptotic Members of Bcl-2 Family is Upregulated, and miR-23a-3p is Downregulated in the Cortex, Hippocampus, and Purified Neurons after Mouse Brain Irradiation

To identify potential mechanisms of neuronal injury after IR exposure, we analyzed the expression of pro-apoptotic members of the Bcl-2 family, such as *Puma* [20], *Noxa* [23], and *Bax* [24] in 30 min, 6 h, 24 h, and 7 days after whole-brain 10 Gy exposure of male C57L/J6 mice. Irradiation caused the up-regulation of *Puma* and *Noxa* mRNAs in cortex 6 h after irradiation. We did not observe changes in the *Bax* mRNA level in the cortex. Irradiation caused the down-regulation of *Bax* in cortex 30 min after irradiation.

**Figure 1:**
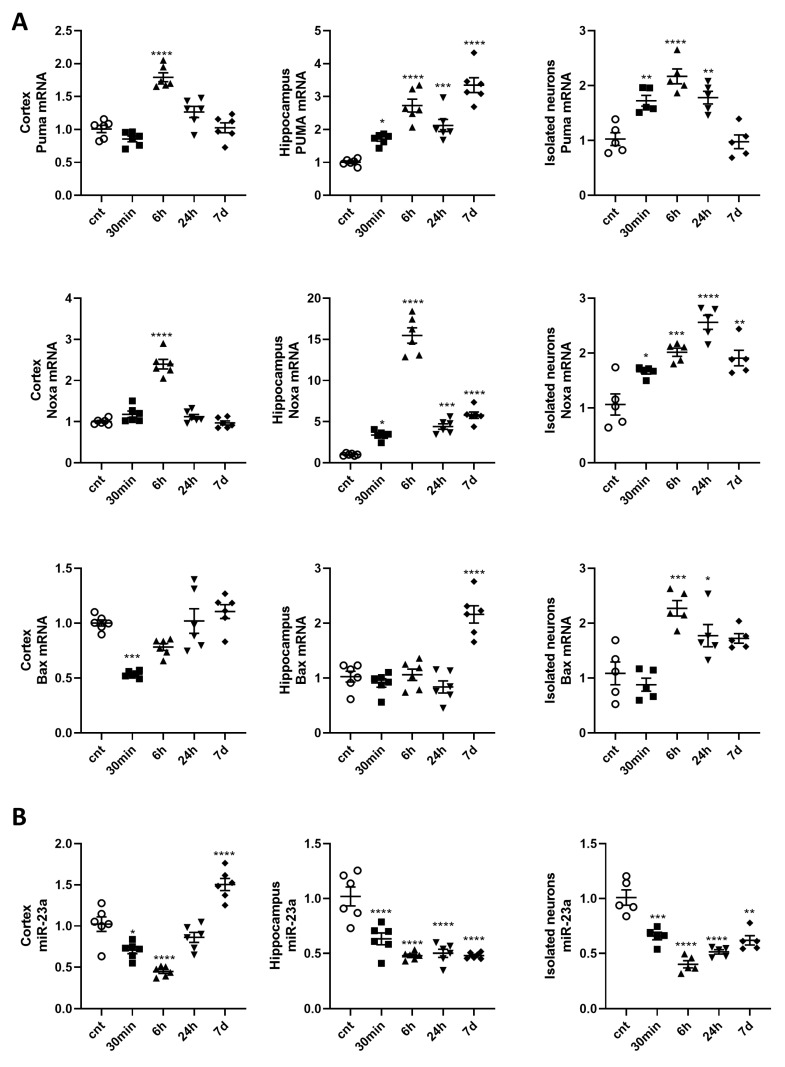
The expression of pro-apoptotic members of the Bcl-2 family is upregulated, and miR-23a-3p is downregulated in the cortex and hippocampus of irradiated mice. Tissues and neurons were collected at 30 min, 6 h, 24 h, and 7 days after 10 Gy whole-brain irradiation. Total RNA was used for qPCR analysis. qPCR quantification of *Puma*, *Noxa*, *Bax* mRNA levels (**A**); and miR-23a-3p (**B**) in cortex, hippocampus, and ex vivo neurons, *n* = 6/group for brain tissues, *n* = 5/group for ex vivo neurons, with two technical replicates. Data represent the mean ± SD of one-way ANOVA and Tukey post-hoc analysis, * *p* < 0.05, ** *p* < 0.01, *** *p* < 0.001, **** *p* < 0.0001 vs. control animals.

IR also induced a rapid and extended increase of *Puma* and *Noxa* mRNA levels in the hippocampus at all time points. The level of Bax mRNA was upregulated in the hippocampus 7 days post-irradiation (Figure 1A).

miR-23a-3p is a validated negative regulator of *Puma*, *Noxa*, and *Bax* expression [20]. We observed a rapid decrease of miR-23a levels in cortex 30 min and 6 h after irradiation and a similar albeit more persistent decrease in the hippocampus at all time points (Figure 1B).

To confirm that the observed changes in gene expression originated from neurons, we isolated neurons from the brain tissues of the same animals. qPCR demonstrated similar changes in the levels of *Puma*, *Noxa*, *Bax*, and miR-23a-3p in isolated brain neurons after 10 Gy whole-brain exposure. Irradiation caused a rapid up-regulation of *Puma* and *Noxa* mRNAs lasting up to 7 days after whole-brain irradiation. *Bax* was up-regulated at 6 h, 24 h, and 7 days after exposure (Figure 1A). We observed a rapid decrease of miR-23a levels in isolated brain neurons at all time points (Figure 1B). Future studies will examine the effects of irradiation on miR-23a-regulated pathways in non-neuronal cell types.

### 2.2. Irradiation Induces Rapid Activation of DNA-Damage and p53 Pathways in Primary Rat Cortical Neurons

**Figure 2:**
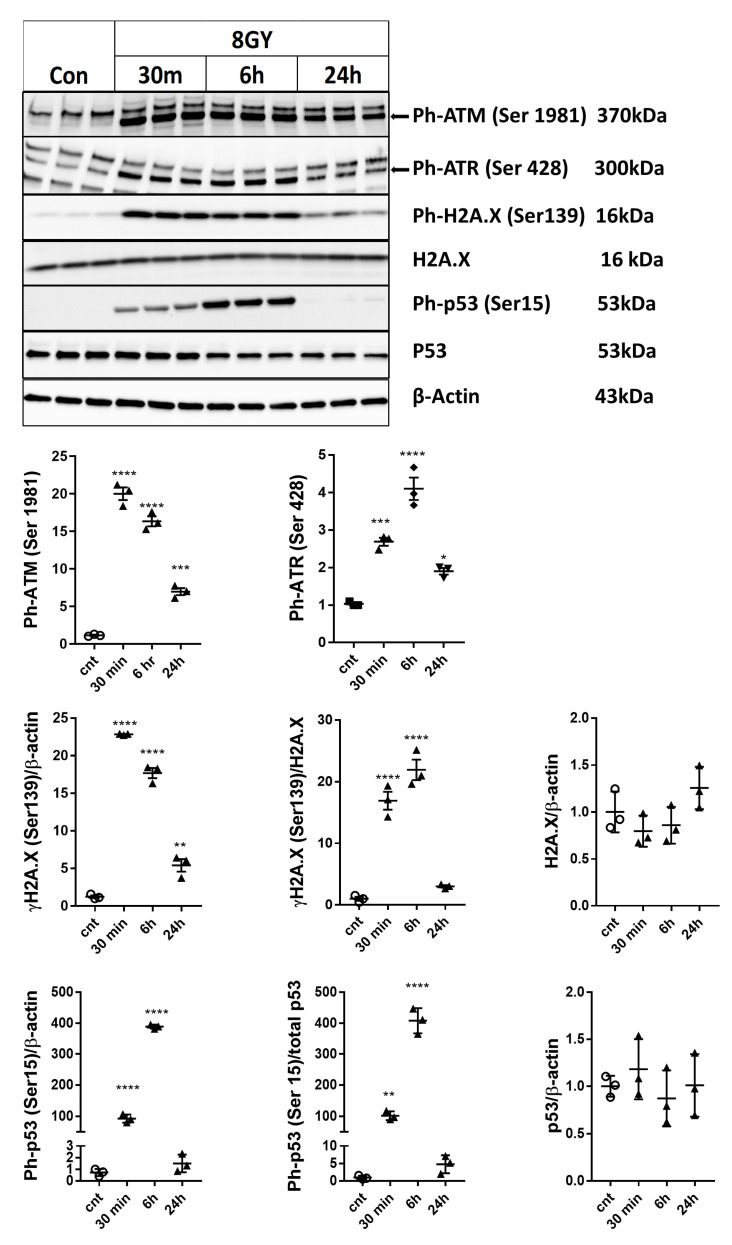
Irradiation induces the activation of DNA damage and p53 pathways in primary cortical neurons. Neurons were collected 30 min, 6 h, and 24 h after 8 Gy irradiation. Image of a representative experiment. Whole-cell lysates were separated by SDS-polyacrylamide gel and immunoblotted with antibodies against Ph-ATM (Ser1981), Ph-ATR (Ser428) (approximately 300kDa), γ-H2A.X (Ser139), and Ph-p53 (Ser15) (representative images). Protein levels were quantified by densitometry; then, they were normalized to β-actin and to the parent protein. Data are presented as fold change compared with untreated control levels. The experiment was repeated 3 times with similar results, *n* = 3/group in each experiment. Data represent the mean ± SD of one-way ANOVA and Tukey post-hoc analysis, * *p* < 0.05, ** *p* < 0.01, *** *p* < 0.001, **** *p* < 0.0001 vs. control RCN.

To investigate whether radiation induced the activation of DNA damage/p53 pathways in neurons, we measured the protein levels of phosphorylated ataxia telangiectasia mutated kinase, Ph-ATM (Ser1981) [25], and Ph-ATR (Ser428) [26], phosphorylated H2A.X (Ser139) (γ-H2A.X) [27], and phosphorylated p53, Ph-p53 (Ser15) [28] proteins by Western blot 30 min, 6 h, and 24 h after 8 Gy irradiation (IR) in comparison to control rat cortical neurons (RCN); total (levels normalized to β-actin) and specific phosphorylation (levels normalized to parent protein) were analyzed, as indicated (Figure 2). For all the investigated mechanisms, the total phosphorylation levels of a given target are what drives the downstream pathways. To quantify total phosphorylation, we normalized the phospho-protein levels to β-actin. In select cases, in which we believed that total protein changes may also occur based on other studies, we examined them separately. An example is p53, as it is known that neuronal injury may cause an elevation of total levels. Thus, for ph-p53 and ph-H2A.X, the levels were normalized to their β-actin (total phosphorylation) and to the total parent proteins. The latter normalization represents a measure of specific phosphorylation and has the additional benefit of indicating the activity levels of upstream regulators. As the lowest dose, which causes significant neuronal cell death (data not shown), the 8 Gy irradiation dose was chosen as the lowest dose, which causes significant neuronal cell death (data not shown). We observed a rapid increase in Ph-ATM (Ser1981) normalized to β-actin; Ph-ATR (Ser428) normalized to β-actin; γ-H2A.X (Ser139) normalized to β-actin; γ-H2A.X (Ser139) normalized to total H2A.X; Ph-p53 (Ser15) normalized to β-actin, and Ph-p53 (Ser15) normalized to total p53. IR did not significantly change the level of total p53 or total H2A.X.

### 2.3. IR Up-Regulates Select Pro-Apoptotic Members of the Bcl-2 Family, Mitochondrial Membrane Permeabilization, and Release of Pro-Apoptotic Molecules in Primary Cortical Neurons

**Figure 3:**
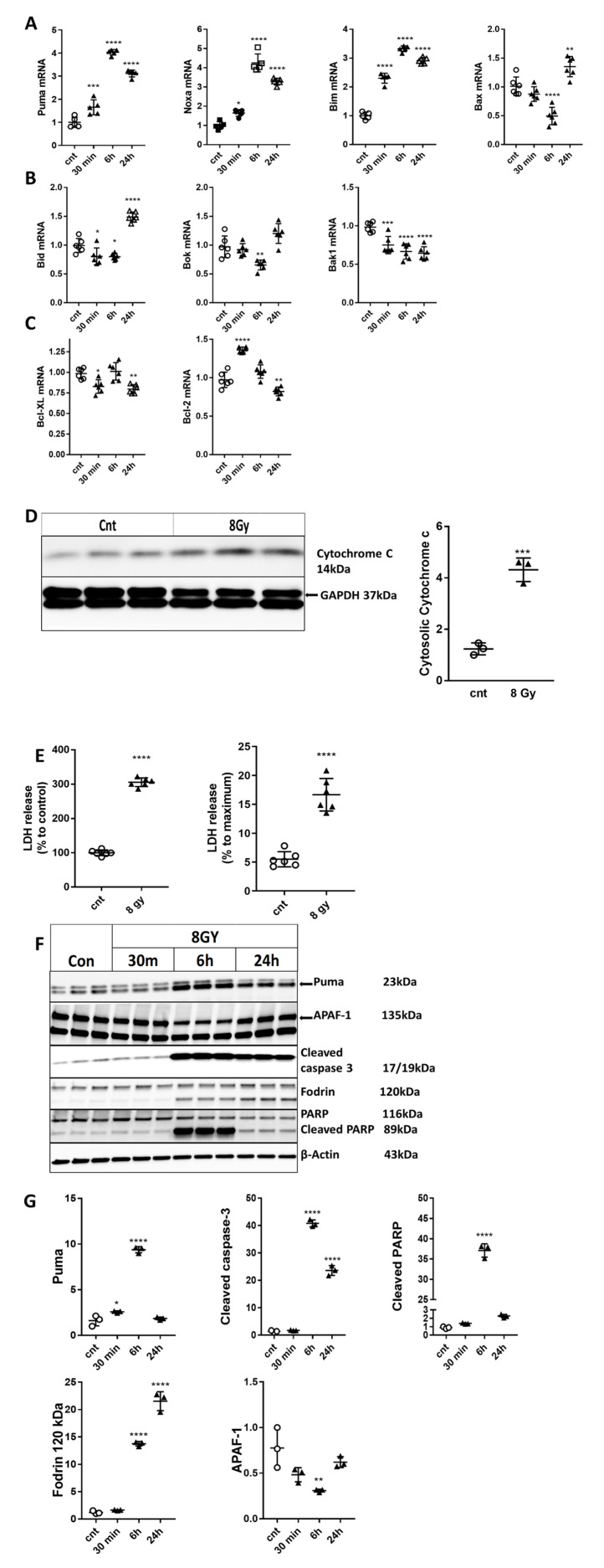
IR up-regulated select pro-apoptotic members of the Bcl-2 family, mitochondrial membrane permeabilization, and release of pro-apoptotic molecules. Neurons were collected at 30 min, 6 h, and 24 h after 8 Gy irradiation. Total RNA was used for qPCR analysis. qPCR quantification of *Puma*, *Noxa*, *Bim*, and *Bax* (**A**); *Bid*, *Bok*, and *Bak1* (**B**), and *Bcl-xL* and *Bcl-2* (**C**) mRNA levels in a representative experiment. The experiment was repeated three times. N = 5/group in each experiment, with two technical replicates. Data represent the mean ± SD of one-way ANOVA and Tukey post-hoc analysis, * *p* < 0.05, ** *p* < 0.01, *** *p* < 0.001, **** *p* < 0.0001 vs. control RCN. RCNs were collected at 24 h after 8 Gy irradiation. Cytosolic fractions were separated by SDS-polyacrylamide gel and immunoblotted with antibodies against cytochrome c and GAPDH. Image of the representative experiment (**D**). Protein levels were quantified by densitometry, normalized to approximately a 37 kDa band of GAPDH, and presented as a fold change compared to control levels. The experiment was repeated 3 times with similar results, *n* = 3/group in each experiment. Data represent the mean ± SD. Statistical significance assigned by one-tailed *t*-test, ***p < 0.001 versus control. Irradiation induces neuronal cell death. Neurons were irradiated with 8 Gy. Twenty-four hours later, LDH release was measured. Data are expressed as a percentage of control untreated neurons as well as completely permeabilized cells (100% cell death) (**E**). The experiment was repeated six times with similar results, *n* = 6/group. Data represent the mean ± SD. Statistical significance was assigned by the one-tailed *t*-test, *p* < 0.0001 vs. control. Rat cortical neurons (RCNs) were collected at 30 min, 6 h, and 24 h after ionizing radiation (IR). Whole-cell lysates were separated by SDS-polyacrylamide gel and immunoblotted with antibodies against Puma, cleaved caspase-3, poly (ADP-ribose) polymerase family, member 1 (PARP), cleaved α-fodrin, and apoptotic peptidase activating factor 1 (Apaf-1). Image of a representative experiment (**F**). Protein levels were quantified by densitometry, normalized to β-actin, and presented as a fold change compared with untreated control levels (**G**). The experiment was repeated three times. *N* = 3/group in each experiment. Data represent mean ± SD of one-way ANOVA and Tukey post-hoc analysis, * *p* < 0.05, ** *p* < 0.01, *** *p* < 0.001, **** *p* < 0.0001 vs. control RCN.

qPCR analysis demonstrated increased gene expression of pro-apoptotic members of the Bcl-2 family, *Puma*, *Noxa*, *Bim* after 8 Gy exposure compared with control samples (Figure 3A). Bax mRNA was down-regulated at 6h and up-regulated at 24 h after IR (Figure 3A). Bid [24] was down-regulated at 30 min and 6 h and up-regulated at 24 h compared to control; Bok [29] was down-regulated at 6 h; Bak1 [30] was modestly but significantly down-regulated after all three time points (Figure 3B). We also examined the expression of pro-survival Bcl2 family members such as *Bcl-2* and *Bcl-xL* [24]. The expression of Bcl-xL was down-regulated at 30 min and 24 h after 8 Gy IR; *Bcl-2* mRNA had a more complex dynamic, being up-regulated at 30 min and down-regulated at 24 h (Figure 3C).

Irradiation results in mitochondrial outer membrane permeabilization (MOMP) [31] with the release of pro-apoptotic molecules [23], including cytochrome c in the cytosolic fraction of IR-treated (8 Gy) primary cortical neurons compared to control cells at 24 h (Figure 3D). Cytochrome c together with apoptotic peptidase activating factor 1 (Apaf-1) forms the apoptosome that triggers the intrinsic caspase activation pathway and caspase-dependent apoptosis [32].

### 2.4. Irradiation Induces Neuronal Cell Death and Time-Dependent Activation of Apoptosis/Caspase-3-Pathways in Primary Cortical Neurons

We observed a significant increase in neuronal cell death (LDH assay) at 24 h after exposure to 8 Gy IR (Figure 3E). Western analysis demonstrated that irradiated neurons displayed increased protein levels of various apoptotic markers (Figure 3F,G). We observed radiation-induced changes in the levels of Puma at 30 min and 6 h. Significant increases were detected for the cleaved/active fragment of caspase-3 at 6 and 24 h as well as for caspase substrates such as cleaved poly (ADP-ribose) polymerase family, member 1 (PARP) at 6 h, and cleaved α-fodrin [32] at 6 and 24 h (Figure 3F,G). Interestingly, IR caused down-regulation of the APAF-1 protein level at 6 h (Figure 3F,G).

### 2.5. miR-23a-3p and -27a-3p were Down-Regulated in Irradiated RCN; Irradiation Decreases Levels of miR-23a-3p, and miR-27a-3p, PUMA and Noxa mRNAs within the RNA-Induced Silencing Complex

**Figure 4:**
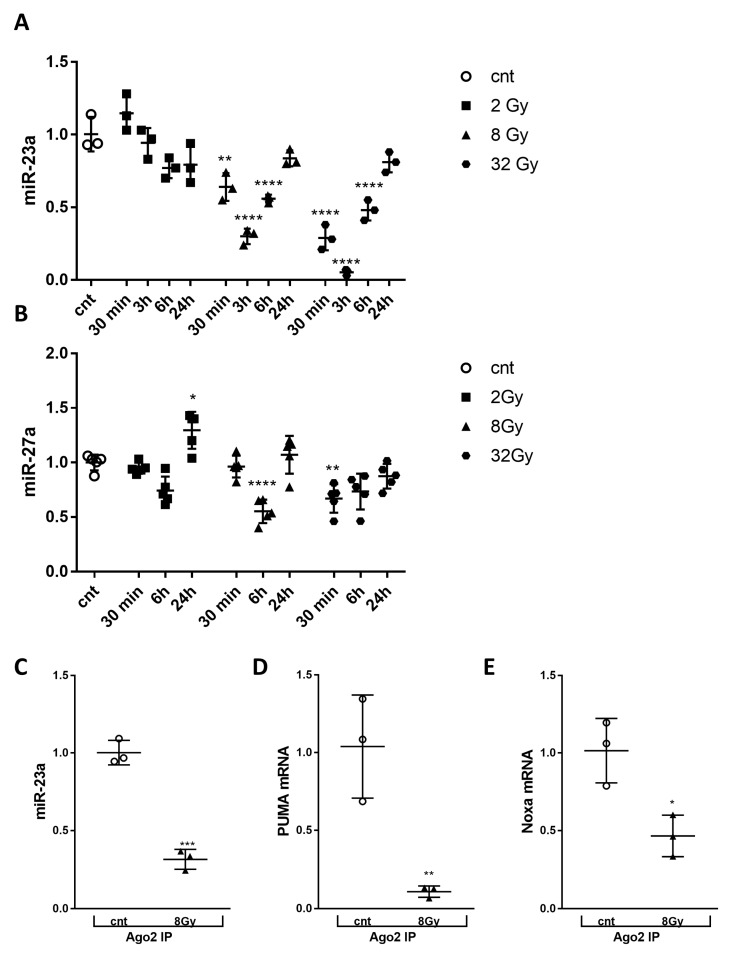
Irradiation decreased levels of cellular miR-23a-3p, and miR-23a-3p, *PUMA*, and *Noxa* mRNAs within the RNA-induced silencing complex. Neurons collected at 30 min, 6 h, and 24 h after 2, 8, and 32 Gy irradiation. Total RNA was used for qPCR analysis. qPCR quantification of miR-23a-3p (**A**) and miR-27a-3p (**B**) levels in a representative experiment. The experiment was repeated three times. N = 3/group in each experiment for miR-23a-3p, *n* = 5/group in each experiment for miR-27a-3p, with 2 technical replicates. Data represent the mean ± SD of one-way ANOVA and Tukey post-hoc analysis * *p* < 0.05, ** *p* < 0.01, *** *p* < 0.001, **** *p* < 0.0001 vs. control RCN. Neurons were collected 3 h after 8 Gy treatment, subjected to RIP with Ago2 antibodies, and samples used for qPCR analysis. qPCR quantification of miR-23a-3p (**C**), Puma (**D**) and Noxa (**E**) levels in precipitates after RIP in a representative experiment. The experiment was repeated two times, *n* = 3/group in each experiment with 2 technical replicates. Data represent the mean ± SD. Statistical significance assigned by one-tailed *t*-test, *** *p* < 0.001 versus control, *n* = 3/group.

We performed a comprehensive expression profile analysis of miR-23a-3p and miR-27a-3p, which are members of the same genomic cluster using qPCR and observed a dose- and time-dependent down-regulation of miR-23a-3p starting as early as 30 min after 8 Gy and 32 Gy treatment and lasting up to 6 h, followed by recovery at 24 h after irradiation (Figure 4A). miR-27a-3p level was also down-regulated at 6 h after 8 Gy and 30 min after 32 Gy (Figure 4B). The down-regulation of miR-23a-3p following irradiation is not reflective of a non-specific response, as other microRNAs were upregulated after IR (data not shown, submitted for publication).

We have previously demonstrated that miR-23a-3p directly targets *Puma* and *Noxa* [20]. miRNA–mRNA target pairs can be purified by the immunoprecipitation of the RISC components to confirm mRNA targets [33]. Here we used RNA-binding protein immunoprecipitation (RIP) with Ago2 antibodies to examine the role of miR-23a-3p in silencing *Puma* and *Noxa* by recruiting these molecules to the RISC after irradiation. RCNs were irradiated with 8 Gy, and cells were collected after 3h (the time point with the highest level of radiation-induced miR-23a-3p down-regulation after 8 Gy) and subjected to RIP using Ago2 antibodies. qPCR using RNA extracted from the Ago2 immunoprecipitate demonstrated a significant and parallel decrease of miR-23a-3p (Figure 4C), Puma mRNA (Figure 4E) in the RISC following 8 Gy treatment.

### 2.6. Transfection with miR-23a-3p Mimic Compensated for the Decrease of Endogenous miR-23a-3p Sequences in Irradiated Primary Cortical Neurons and Attenuates Neuronal Cell Death

**Figure 5:**
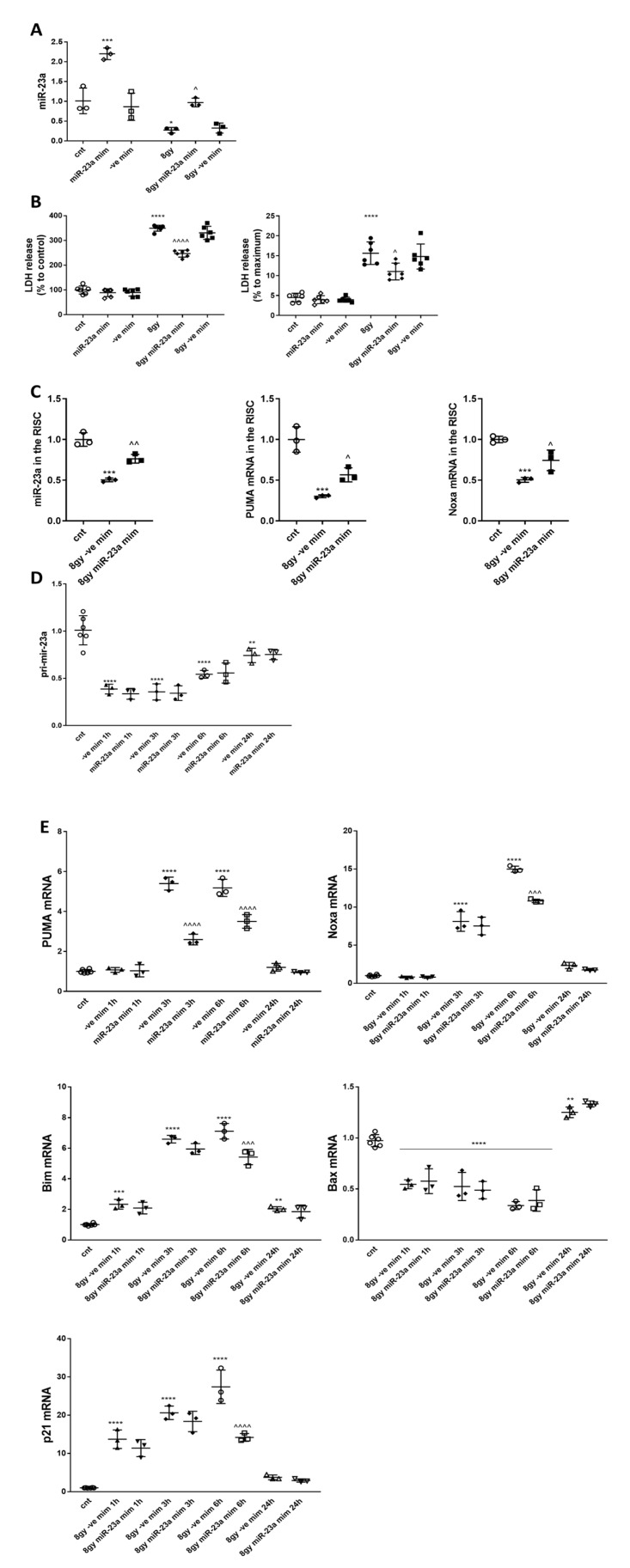
miR-23a-3p mimic reverses the decrease of endogenous miR-23a-3p in irradiated primary cortical neurons and attenuates neuronal cell death. RCNs were transfected with miR-23a-3p mimics and negative control mimics before irradiation. At 3 h after exposure to 8 Gy, neurons were harvested for RNA isolation. qPCR quantification of miR-23a-3p in a representative experiment (**A**). The experiment was repeated two times. N = 3/group in each experiment with 2 technical replicates. Data represent mean ± SD. Statistical significance assigned by one-way ANOVA and Tukey post hoc analysis; * *p* < 0.05, *** *p* < 0.001 vs. control RCN; ^ *p* < 0.05 vs. irradiated negative control mimics. LDH release was measured at 24 h after irradiation as a percentage of control untreated neurons as well as completely permeabilized cells (100% cell death). Analysis of a representative experiment (**B**). The experiment was repeated four times with similar results, *n* = 6/group in each experiment. Data represent mean ± SD. Statistical significance assigned by one-way ANOVA and Tukey post hoc analysis, * *p* < 0.05, *** *p* < 0.001, vs. control RCN. ^ *p* < 0.05, ^^^^ *p* < 0.0001 vs. negative control mimic transfected cells (-ve mim) RCN. RCNs were treated as described above and harvested 3 h after 8 Gy irradiation. Lysates from each sample were subjected to RNA-binding protein immunoprecipitation (RIP) using Ago2 antibodies, followed by qPCR analysis for levels of miR-23a-3p, Puma, and Noxa in the RISC. Analysis of a representative experiment (**C**) *n* = 3/group in each experiment with 2 technical replicates. Data represent mean ± SD. Significance assigned by one-way ANOVA and Tukey post hoc analysis, miR-23a-3p *** *p* = 0.0001 vs. control RCNs; ^^ *p* < 0.0039 vs. 8 Gy + miR-ve mimic; *Puma* *** *p* = 0.0004 vs. control RCNs; ^ *p* < 0.0471 vs. 8 Gy + miR-ve mimic; *Noxa* *** *p* = 0.0006 vs. control RCNs; ^ *p* < 0.0228 vs. 8 Gy + miR-ve mimic. IR causes the down-regulation of miR-23a-3p at the transcription stage. qPCR quantification of pri-miR-23a-3p. Analysis of a representative experiment (**D**). Data represent the mean ± SD of one-way ANOVA and Tukey post-hoc analysis, *n* = 6 for the control group, *n* = 3 for all other groups with 2 technical replicates in each experiment; * *p* < 0.05, ****p < 0.0001 vs. control RCN. miR-23a-3p mimic attenuates the irradiation-induced elevation of *Puma*, *Noxa*, and *Bim* in primary cortical neurons. RCNs were transfected with miR-23a-3p mimics and negative control mimics before irradiation. Neurons were harvested for RNA isolation at 1 h, 3 h, 6 h, and 24 h after exposure to 8 Gy. qPCR quantification of *Puma*, *Noxa*, *Bim*, and *Bax* and *p2*1. Analysis of a representative experiment (**E**). The experiment was repeated three times, *n* = 6 for control, *n* = 3 for all other groups in each experiment with 2 technical replicates. Data represent mean ± SD. Statistical significance assigned by one-way ANOVA and Tukey post hoc analysis, ** *p* < 0.01, *** *p* < 0.001, **** *p* < 0.0001 vs. control RCN, ^^^ *p* < 0.001, ^^^^ *p* < 0.0001 vs. negative control mimic transfected cells (-ve mim) RCN.

To test the hypothesis that miR-23a-3p decline plays a significant role in radiation-induced neuronal cell death, we transfected primary cortical neurons with miR-23a-3p and negative control mimics before irradiation. 3 h after exposure to 8 Gy IR, neurons were harvested for RNA isolation and qPCR for miR-23a-3p. The total level of miR-23a-3p in neurons transfected with the miR-23a-3p mimic (representing endogenous miR-23a plus the transfected miR-23a mimic) was 2 times higher than in control cells; IR induced the down-regulation of miR-23a-3p. The level of miR-23a-3p in irradiated neurons transfected with the miR-23a-3p mimic (representing remaining endogenous miR-23a plus the transfected miR-23a mimic) was similar to the level in nonirradiated RCNs and significantly higher than in irradiated neurons transfected with the negative control mimic, attenuating the radiation-induced decline in endogenous miR-23a-3p (Figure 5A). Transfection with negative control mimics did not change the level of miR-23a-3p. Importantly, the attenuation of the miR-23a-3p decline by transfection with miR-23a-3p mimic significantly reduced irradiation-induced neuronal cell death (LDH assay) at 24 h after 8 Gy treatment compared to neurons transfected with negative control (Figure 5B).

The irradiation-induced decrease of miR-23a-3p, *PUMA*, and *Noxa* mRNAs within the RNA-induced silencing complex was attenuated by miR-23a-3p mimic compared to miR-ve control mimic. We detected a significant down-regulation of miR-23a-3p and its targets, *PUMA* and *Noxa* in the RISC after IR in RCNs transfected with miR-ve mimic compared to controls. miR-23a-3p mimic attenuated the IR-induced down-regulation of miR-23a-3p, *Puma*, and *Noxa* in the RISC compared to miR-ve mimic (Figure 5C).

The level of pri-miR-23a (a precursor of miR-23a) was analyzed to investigate the mechanisms of IR-induced down-regulation of mature miR-23a-3p. RCNs were transfected with miR-23a-3p and negative control mimics before irradiation (8 Gy) and collected at 1 h, 3 h, 6 h, and 24 h after irradiation. We observed a rapid decrease in pri-miR-23a levels (a marker of miR-23a-3p transcription), with the lowest levels at 1–3 h and progressively returning toward control levels at 6 h and 24 h. miR-23a-3p and negative control mimics had no effect on pri-mir-23a levels (Figure 5D).

### 2.7. miR-23a-3p Mimic Attenuates Irradiation-Induced Elevation of Puma, Noxa, and Bim in Primary Cortical Neurons

We also investigated the effect of transfection with miR-23a-3p mimic on the expression of key pro-apoptotic members of the Bcl-2 family in neurons exposed to irradiation (8 Gy). Levels of *Puma*, *Noxa*, *Bim*, and *Bax* mRNA were analyzed by qPCR, demonstrating that the miR-23a-3p mimics significantly attenuated the radiation-induced increase in *PUMA* at 3 and 6 h after 8 Gy IR, *Noxa* at 6 h after IR, and *Bim* at 6 h after IR compared to irradiated neurons transfected with negative control mimic (Figure 5E). *Bax* mRNA was rapidly down-regulated at 1 h, 3 h, and 6 h after IR and up-regulated at 24 h compared to control. miR-23a-3p mimic did not affect Bax mRNA levels after irradiation compared to irradiated RCNs transfected with negative control mimics (Figure 5F). We also investigated the effect of transfection with miR-23-3p mimic on the expression of the p53 downstream target *CDKN1A-(p21CIP1/WAF1) (p21)* [34]. qPCR demonstrated that IR rapidly upregulated *p21* expression at 30 min and 6 h after 8 Gy IR. miR-23a-3p mimic significantly attenuated the radiation-induced increase in *p21* expression at 6 h after 8 Gy IR (Figure 5E).

### 2.8. miR-23a-3p Mimic Attenuates DNA Damage Response and p53 Activation in Primary Cortical Neurons Following Irradiation

**Figure 6:**
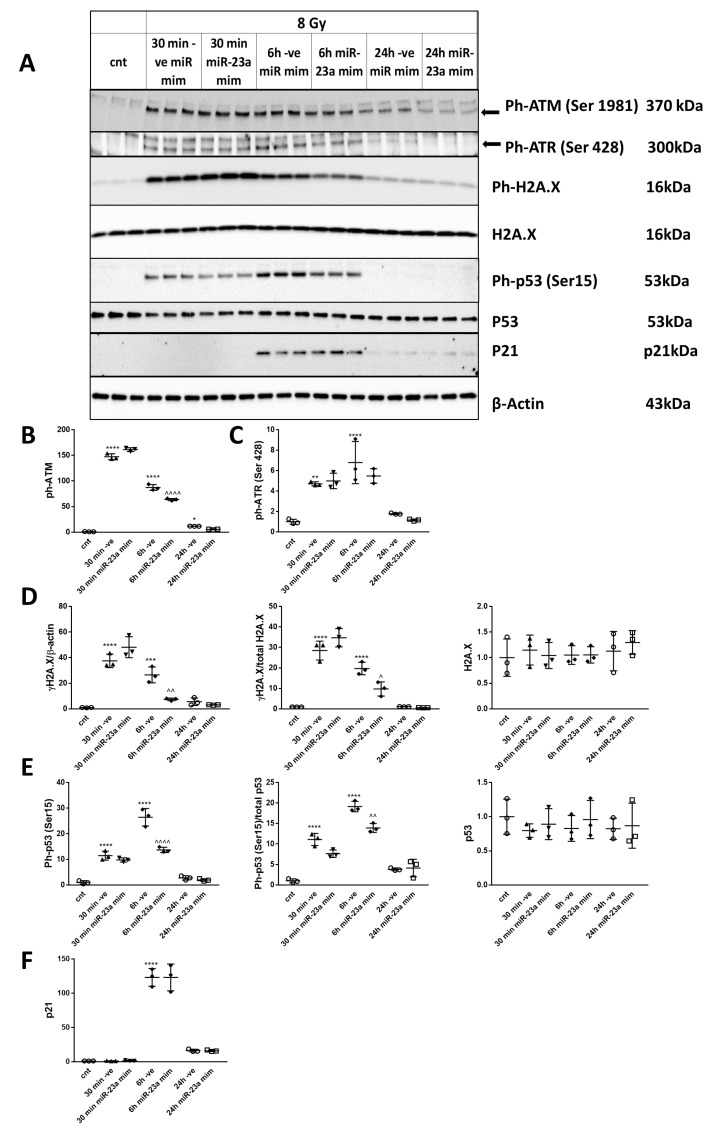
miR-23a-3p attenuates DNA damage response and p53 activation in primary cortical neurons following irradiation. RCNs were transfected with miR-23a-3p mimics, and negative control mimics before irradiation. Neurons were collected at 30 min, 6 h, and 24 h after 8 Gy irradiation. Whole-cell lysates were separated by SDS-polyacrylamide gel and immunoblotted with antibodies against Ph-ATM (Ser1981), Ph-ATR (Ser428) (approximately 300 kDa), γ-H2A.X (Ser139), H2A.X, Ph-p53 (Ser15), p53, p21 and β-actin (representative image). Image of a representative experiment (**A**). Protein levels were quantified by densitometry, normalized to β-actin and the parent proteins for γ-H2A.X and Ph-p53, and presented as fold change compared with untreated control levels (**B**–**F**). The experiment was repeated 3 times with similar results, *n* = 3/group in each experiment. Data represent mean ± SD. Statistical significance assigned by one-way ANOVA and Tukey post-hoc analysis, * *p* < 0.05, ** p < 0.01, *** p < 0.001, **** *p* < 0.0001 vs. control RCN, ^^ *p* < 0.01, ^^^^ *p* < 0.0001 vs. negative control mimic transfected cells (-ve mim) RCN.

RCNs were transfected with miR-23a-3p and negative control mimics before irradiation (8 Gy) to examine the effect of miR-23a-3p mimic on DNA damage response and p53 activation. We analyzed the levels of Ph-ATM (Ser1981), Ph-ATR (Ser428), γ-H2A.X (Ser139), Ph-p53 (Ser15), and p21 by Western blot (Figure 6A) at 30 min, 6 h, and 24 h after 8 Gy irradiation. IR caused DNA damage and rapid increases in the levels of Ph-ATM (Ser1981) (Figure 6B), Ph-ATR (Ser428) (Figure 6C), γ-H2A.X (Ser139) at 30 min and 6 h-normalized to β-actin and to total H2A.X and Ph-p53 at 30 min and 6 h-normalized to β-actin and to total p53 (Figure 6D). The levels of all phospho-proteins returned toward normal by 24 h after IR treatment and were not significantly different from control levels (Figure 6B–E). Treatment with miR-23a-3p mimic attenuated the increased phosphorylation for Ph-ATM (Ser1981) (Figure 6A,B), γ-H2A.X, and Ph-p53 at the 6h time point (Figure 6A,D). Neither IR nor miR-23a-3p mimics changed the levels of total H2A.X and p53 (Figure 6A,D,E) normalized to β-actin. qPCR analysis also demonstrated that IR did not change the levels of p53 mRNA (data not shown). IR also up-regulated the level of p53’s downstream target p21 at 6 h after irradiation. Treatment with miR-23a-3p mimic did not attenuate the increase in p21 protein levels (Figure 6A,F).

### 2.9. miR-23a-3p Attenuates DNA Damage Markers in Primary Cortical Neurons Following Irradiation

**Figure 7:**
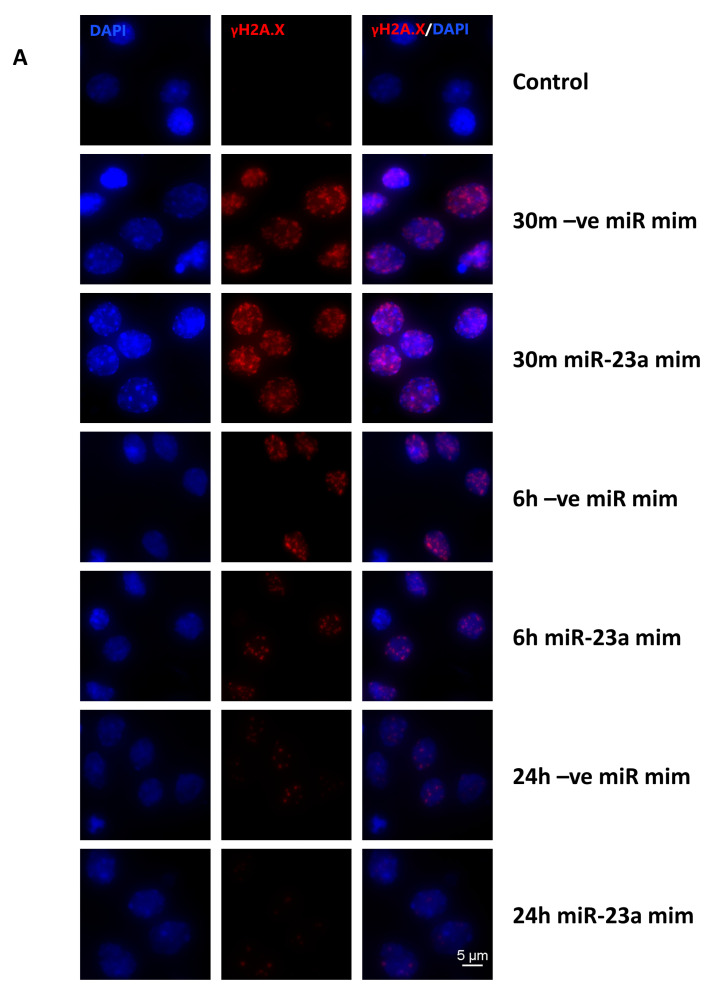

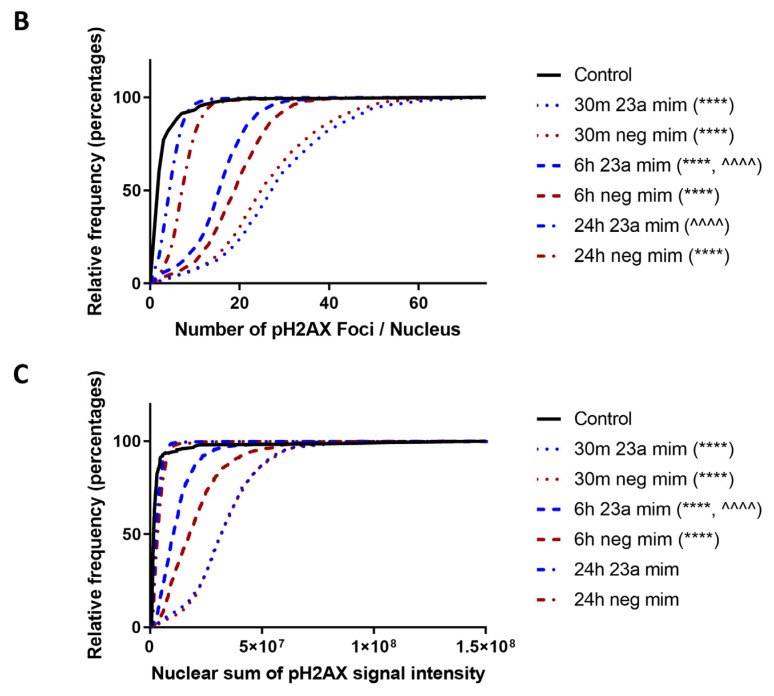
miR-23a-3p attenuates DNA damage response in primary cortical neurons following irradiation. Representative microscopy images from 30 min, 6 h, and 24 h of RCNs stained for γ-H2A.X (red), and 4′,6-diamidino-2-phenylindole (DAPI) (blue) (**A**). Data were calculated and plotted for all fields together as a cumulative frequency distribution without binning for γ-H2A.X foci count (**B**) and nuclear staining intensity (**C**). Eight fields were acquired per treatment as detailed above, and an average of 117 nuclei were identified per field.

We performed a quantitative analysis of γH2A.X immunocytochemistry and evaluated the effect of miR-23a-3p mimic on accumulated DNA damage by measuring γH2A.X foci formation and progression after 8 Gy irradiation. IR significantly increased γH2A.X foci number at all time points as well as signal intensity per nucleus (Figure 7A–C) at 30 min and 6 h in miR-ve mimic samples compared to the non-irradiated control, shifting the cell population signal intensity distribution curve to the right; these changes were progressively attenuated with time. miR-23a-3p mimic led to a significant decrease in both parameters at 6 h and in foci number at 24 h compared to miR-ve mimic, pushing the cell population distribution curve to the left.

### 2.10. miR-23a-3p Mimic Attenuates MOMP and Caspase-Dependent Neuronal Apoptosis after Irradiation

**Figure 8:**
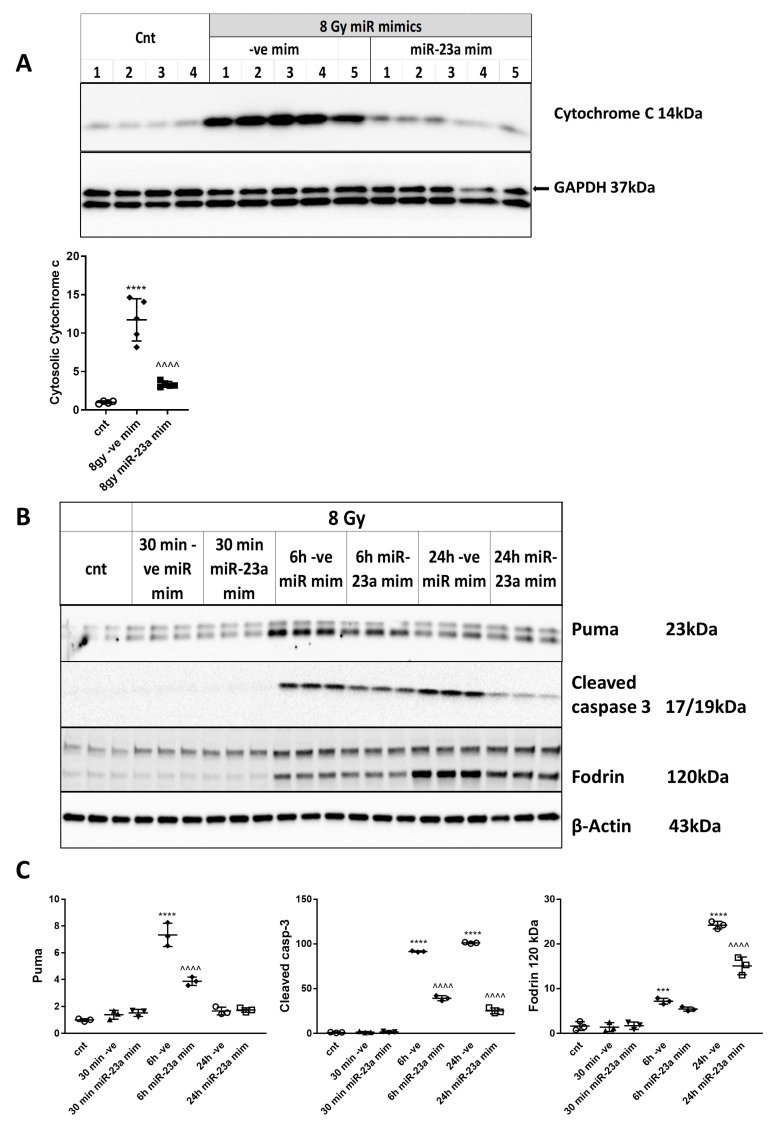
miR-23a-3p attenuates caspase-dependent neuronal apoptosis after irradiation. Neurons were transfected with miR-23a-3p mimics and miR-ve mimics and collected at 6 h after 8 Gy irradiation. Cytosolic fractions were separated by SDS-polyacrylamide gel and immunoblotted with antibodies against cytochrome c and GAPDH. Image of a representative experiment (**A**). Protein levels were quantified by densitometry, normalized to an approximately 37 kDa band of GAPDH, and presented as a fold change compared to control levels, *n* = 4 for control, *n* = 5 for other groups in each experiment. The experiment was repeated three times. Data represent mean ± SD. Significance assigned by one-way ANOVA and Tukey post-hoc analysis, **** *p* < 0.0001 vs. control RCNs; ^^^^ *p* < 0.0001 vs. 8 Gy + miR-ve mimic. RCNs were transfected with miR-23a-3p mimics, and negative control mimics before irradiation. Neurons were collected at 30 min, 6 h, and 24 h after 8 Gy irradiation. Whole-cell lysates were separated by SDS-polyacrylamide gel and immunoblotted with antibodies against Puma, α-fodrin, and cleaved caspase-3. Image of a representative experiment. (**B**). Protein levels were quantified by densitometry, normalized to β-actin, and presented as fold change compared with untreated control levels (**C**). The experiment was repeated 3 times with similar results, *n* = 3/group in each experiment. Data represent mean ± SD of one-way ANOVA and Tukey post hoc analysis, *** *p* < 0.001, **** *p* < 0.0001 vs. control RCN, ^^^^ *p* < 0.0001 vs. negative control mimic transfected cells (-ve mim) RCN.

Subcellular fractionation revealed that 8 Gy of IR caused the release of cytochrome c from the mitochondria into the cytosol at 6h in RCNs transfected with negative control mimics compared to control RCNs (Figure 8A). miR-23a-3p mimic attenuated the IR-induced mitochondrial release of cytochrome c compared to the miR-ve control mimic (Figure 8A). RCNs were transfected with miR-23a-3p and negative control mimics before irradiation (8 Gy) and examined at 30 min, 6 h, and 24 h after irradiation. Western blot demonstrated that neurons transfected with miR-23a-3p mimic displayed reduced levels of markers of apoptosis (Figure 8B,C), including Puma at 6 h, cleaved (active) fragment of caspase-3 at 6 and 24 h, and cleaved α-fodrin (120 kDa) at 24 h after irradiation compared to neurons transfected with negative control mimics (Figure 8B,C).

## 3. Discussion

In contrast to the better-defined responses triggered by irradiation in tumor cells, much less is known about the apoptotic mechanisms activated in neurons following IR-exposure. There are two novel elements of the described research. (1) First, we demonstrate that irradiation triggers intrinsic apoptosis in primary neurons in vitro including the activation of DNA-damage responses, followed by p53-dependent up-regulation of the pro-apoptotic Bcl-2 family molecules PUMA, Noxa, and Bim, mitochondrial outer membrane permeabilization, and the release of cytochrome c, which activates intrinsic caspase-dependent apoptosis. Meanwhile, our study has focused on demonstrating the role of miR-23a down-regulation in the development of intrinsic apoptosis after neuronal irradiation and did not prove the causal links between each downstream step in the indicated cascade; the latter were previously established by multiple other studies [23,35]. (2) We demonstrate that miR-23a-3p, a key negative regulator of pro-apoptotic Bcl-2 family molecules, rapidly decreases after neuronal irradiation in vitro, which may contribute to the secondary elevation of its targets PUMA and Noxa. Moreover, the administration of a miR-23a-3p mimic inhibits the irradiation-induced up-regulation of Noxa and Puma via the degradation of PUMA and Noxa mRNAs in the RNA-induced silencing complex, significantly attenuates intrinsic apoptosis, and reduces neuronal cell death. The neuroprotective effects of miR-23a-3p administration may also include the attenuation of secondary DNA damage upstream of p53 activation. (3) Third, we demonstrate that miR-23a-3p down-regulation and key elements of the intrinsic apoptotic machinery are present after X-ray exposure of the brain in vivo. Thus, brain irradiation in vivo results in pro-apoptotic molecular changes in the cortex and hippocampus, including the down-regulation of miR-23a-3p and elevation of pro-apoptotic Bcl2-family molecules PUMA, Noxa, and Bax in the hippocampus. Importantly, using cell-specific purification, we show that these changes are present in isolated neurons from the irradiated mouse brains.

The use of primary neurons an in vitro model permitted a mechanistic investigation to elucidate the progression of IR-induced cell death mechanisms. IR caused neuronal DNA damage followed by the sequential phosphorylation/activation of ATM(Ser1981)/ATR(Ser428) and phosphorylation of histone H2A.X(Ser128) as well as phosphorylation/activation of p53 at Ser15, which is necessary for the transactivation of pro-apoptotic Bcl-2 family members [28] and p21 [36]. This is followed by the downstream activation of p53-mediated cell death pathways involving p53-dependent transcriptional activation of pro-apoptotic Bcl-2 family members such as BH3-domain-only Puma, Noxa, and Bim [37]. Other pro-apoptotic Bcl-2 family molecules, including Bid, Bax, Bok, and Bak as well as anti-apoptotic Bcl-2 family members such as Bcl-xL and Bcl-2, also undergo expression changes after IR and may contribute to the irradiation-induced imbalance between neuronal survival and apoptotic pathways in a manner that favors the latter.

The up-regulation of BH3-only molecules leads to MOMP with the release of pro-apoptotic cytochrome c from the mitochondria into the cytosol, where together with Apaf-1, it forms the apoptosome and triggers caspase-dependent apoptosis. We confirmed that IR induces the release of cytochrome c from the mitochondria to the cytosol, the activation of caspase-3, and the cleavage of PARP and α-fodrin, which are markers of the execution phase of neuronal apoptosis [32].

miRs have been implicated in CNS disorders [16,17,18] acting as modulators of neuronal cell death pathways [19]. The levels of several miRNAs are altered in non-neuronal cells after irradiation [38], including the focal brain irradiation-induced up-regulation of miR-7 in astrocytes and oligodendrocytes [39] and whole-body IR exposure-induced changes in miRs levels in the cortex and hippocampus of neonatal mice [40,41,42]. However, these data have not revealed the effects of irradiation on neuronal miRs levels and the impact of these changes on IR-induced neuronal cell death. Here, for the first time, we demonstrated the down-regulation of miR-23a expression in neurons isolated from the brain following IR exposure.

The involvement of miRs in IR-induced DNA damage and neuronal cell death is supported by studies indicating that miRs may regulate oxidative stress-induced neuronal cell death [43,44]. Reduced levels of miR-23a-3p/27a after traumatic brain injury (TBI) may reflect neuronal DNA damage in response to oxidative stress and were shown to contribute to a post-traumatic neuronal loss [20].

In the present studies, neuronal IR caused a dose-dependent and transient down-regulation of miR-23a-3p and miR-27a, which are members of the same genomic cluster that are expressed as a single primary transcript [20]. Pri-mir-23 levels (an indicator of microRNA transcription) [45] were down-regulated after IR, suggesting that miR-23a-3p decline is, at least in part, mediated via rapid transcriptional inhibition. We previously demonstrated that miR-23a-3p inhibits Puma and Noxa expression by directly targeting their 3′ UTRs [20]. Here, we show that irradiation leads to a reduction in miR-23a-3p and its pro-apoptotic targets Noxa and PUMA in the neuronal RISC complex. Thus, an IR-mediated decrease in cellular miR-23a-3p reduces the recruitment of Puma and Noxa mRNAs to the RISC complex and consequently increases Puma and Noxa neuronal levels. Importantly, the administration of miR-23a-3p mimics reverses these changes, attenuating IR-induced MOMP and the release of cytochrome c as well as decreasing caspase-3 pathway activation and neuronal cell death. Our data support a model in which IR-mediated down-regulation of miR-23a-3p acts independently of p53-dependent transcription to enhance the up-regulation of Noxa and PUMA mRNAs levels, strengthening the activity of apoptotic pathways and promoting neuronal cell death.

Pro-apoptotic molecules Bax and Apaf-1 [46], as well as HIF-1α [45], are other predicted targets of miR-23a, but no corresponding changes were observed after neuronal IR (data not shown), which is suggestive of model-specific differences.

Our in vitro studies uncovered the transient character of virtually all observed molecular mechanisms, with DNA damage/repair, miR-23a-3p, and apoptotic mechanisms demonstrating a rapid “activation” phase, reaching a maximum amplitude of changes before 6 h followed by a significant decline toward 24 h, when the cell fate has already been decided. This dynamic profile suggests that neuronal IR elicits parallel repair and cell death pathways, and the ultimate outcome is determined stochastically across the cell population based on the individual balance achieved. An excessive activation of cell death mechanisms could decrease the opportunity for repair mechanisms to mend the damage. Blocking the endogenous decline in miR-23a-3p by the administration of the mimic shifts the balance by attenuating apoptotic mechanisms and ultimately increases neuronal survival.

**Figure 9:**
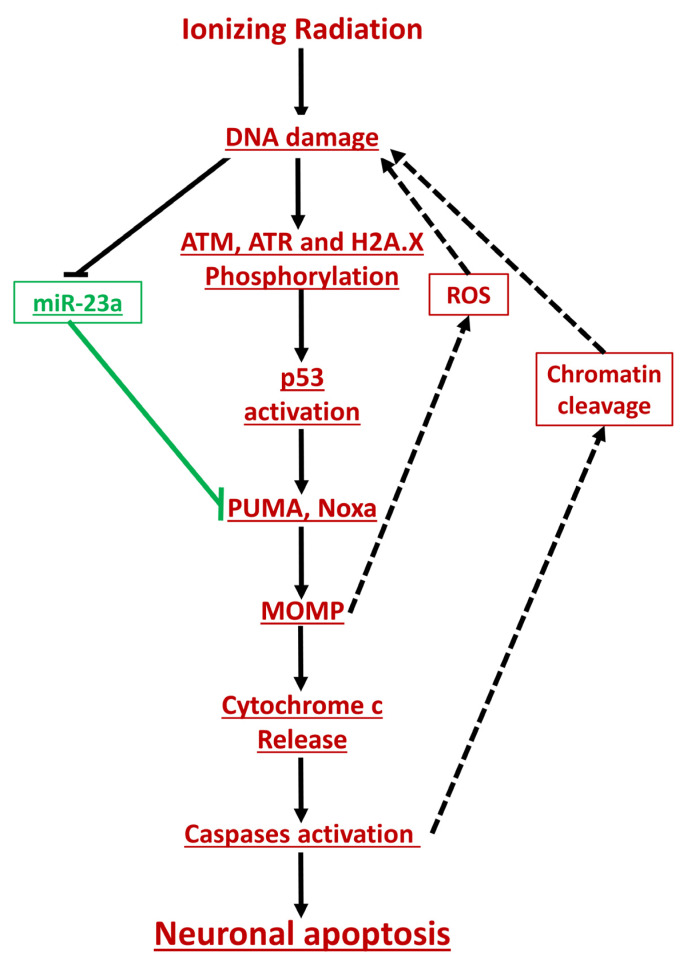
The schematic illustration of the role of miR-23a on the IR-induced neuronal outcome. Pro-apoptotic events are shown in red, anti-apoptotic events are shown in green. Confirmed changes are underlined and shown in solid arrows.

Interestingly, the administration of miR-23a-3p mimics also appeared to have accelerated the clearing of DNA damage (decreased ATM phosphorylation, γH2AX) and attenuated p53 phosphorylation/activation. No changes in total p53 levels were observed in response to miR-23a-3p mimic, suggesting that p53 is not a direct target of miR-23a-3p. Nonetheless, the miR-23a-3p mimic-dependent attenuation of p53 transcriptional activity was demonstrated by the decreased expression of p21 mRNA, which is a well-known p53 target gene and neuronal apoptosis regulator [34]; no parallel attenuation of p21 protein levels was observed, suggesting the existence of transcription-independent regulatory mechanisms [47,48,49]. The observed inhibition of ATM and histone phosphorylation was detected at 6 h and 24 h but not at 30 min after irradiation, suggesting it involved secondary processes and not modulation of the original DNA damage. γH2AX accumulation and especially the formation of nuclear γH2AX foci is a sensitive marker of DNA damage [50], and the progressive decline in γH2AX foci uncovered by the quantitative examination of immunocytochemistry across the cell population reflects the efforts of DNA repair processes to clear the primary and secondary DNA damage. Our data showing a miR-23a-3p-dependent reduction in γH2AX foci at 6 h and 24 h but not 30 min post-IR suggest that the miR-23a mimic may attenuate the secondary post-IR accumulation of DNA damage without limiting the initial injury. This interpretation is consistent with studies that showed that apoptotic DNA fragmentation induces γH2AX formation [51] and may lead to p53-dependent changes in gene expression to further apoptosis [52]. Moreover, mitochondrial depolarization increases reactive oxygen species (ROS) production [53,54], producing more DNA damage. Thus, the miR-23a-dependent down-regulation of Puma and Noxa and the attenuation of MOMP may reduce the accumulation of ROS, secondary DNA damage, and additional p53 activation. Conversely, miR-23a-3p-mimic inhibits this positive feedback loop by which apoptosis-induced chromatin cleavage adds to the total DNA damage and amplifies apoptosis. Thus, the specific attenuation of p53 activation suggests that miR-23a-3p administration reduces neuronal apoptosis after IR not only through the direct down-regulation of Puma and Noxa but also through the modulation of mechanisms upstream of p53 (Figure 9).

Key components of the molecular mechanisms activated by IR in primary neurons in vitro are also present in the brain after irradiation in vivo. Our data show that cortical responses demonstrate a similar temporal profile in regard to miR-23a-3p and BH3-only molecules changes with rapid down- and up-regulation, respectively and return to toward normal by 24 h. The hippocampus shows a markedly distinct dynamic in which changes are more persistent and last as long as 7 days post-IR. Significantly, we also uncover the IR-induced molecular changes in isolated neurons (from generally sub-cortical areas), which are found to most resemble the hippocampus pattern. While the profile of changes miR-23a and key apoptotic pathways display important similarities across the studied in vivo and in vitro models and brain regions/neuronal populations, the observed differences may be explained by the presence of non-neuronal cells as well as neuronal subtype-specific effects. Thus, the persistent miR-23a down-regulation in the hippocampus may reflect the presence of neural progenitors and developing immature neurons with higher radiosensitivity [55,56]. Future studies will examine the miR-23-regulated mechanisms across various cell types and neuronal sub-types, including the specific responsiveness of irradiation-induced cell death to miR-23 modulation. Clearly, the detection of characteristic regional/cellular changes indicates the need for future studies to generate a more complete profile of neuronal cell death processes after IR in vivo and explore the effects of miR-23a-3p targeting.

## 4. Materials and Methods

### 4.1. Animals and Radiation Delivery and Quality Control

Male C57L/J6 mice (Jackson Labs, Bar Harbor, ME, USA) at approximately 10–12 weeks weighing ≥ 20 g were anesthetized by i.p. injection of 80–100 mg·kg^−1^ ketamine and 10–15 mg·kg^−1^ xylazine 15 min prior to radiation exposure to prevent them from moving out of the field. C57L/J mice were exposed to 10 Gy of 320 kVp X-rays to the whole-brain (1.25 Gy·min^−1^, HVL = 1 mm Cu, Pantak 320 X-ray Irradiator, Precision X-ray, Precision X-ray, Inc., North Branford, CT, USA). A calibration quality check was performed each morning and again in between each radiation run. Port films (Gafchromic EBT2 dosimetry film, Ashland, Bridgewater, NJ, USA) were acquired during each radiation run to verify that the brains of each mouse were properly positioned within the radiation field. All experiments were conducted in compliance with the Animal Use Protocol approved by the Institutional Animal Care and Use Committee.

### 4.2. In Vitro Cell Culture

Primary neuronal cultures were used in this study to identify neuron-specific responses. RCNs were derived from rat (Sprague Dawley, supplier: Envigo, Indianapolis, IN, USA) embryonic cortices (as previously described) [20]. Neurons were maintained in serum-free conditions using the B-27 Plus Supplement (Thermo Fisher Scientific, Waltham, MA, USA) according to the manufacturer’s protocol. The transfection of RCNs was performed at seven days in vitro. RCNs were transfected with miRIDIAN rat miR-23a-3p mimic (C-320309-03-0005) or miRIDIAN microRNA Mimic Negative Control (CN-001000-01-05) (Dharmacon) using the Lipofectamine RNAiMAX Transfection Reagent (Invitrogen, Life Technologies, Carlsbad, CA, USA) according to the manufacturer’s protocol. The sequence of miRIDIAN microRNA Mimic Negative Control is based on Caenorhabditis elegans microRNAs and have minimal sequence identity in human, mouse, and rat. Based on titration experiments, we chose a final concentration of 50 nM for the miR mimics and negative control mimics. This concentration resulted in optimal transfection efficiency (approximately 50%), was devoid of nonspecific changes in non-targeted miRs, and had no neurotoxic effects [20]. One hour after transfection, the media was replaced with normal conditioned media.

### 4.3. RCNs Irradiation

After culturing for seven days, in vitro RCNs were exposed to X-rays using a PANTAK SEIFRT X-RAD X-ray System (model number HS320, Precision X-ray, Inc., North Branford, CT, USA) with energy settings 250 KeV and 13 mA. For most of the experiments, we used an 8 Gy dose. Based on our experiments (data not shown), the 8 Gy is the lowest dose, which causes significant neuronal cell death.

### 4.4. Cell Death Assays

Terminal deoxynucleotidyl transferase dUTP nick end labeling (TUNEL) staining is not a reliable method to examine apoptotic DNA fragmentation in this model, as it is confounded by IR-induced DNA double-strand breaks [23]. Cell death was measured using the CytoTox 96^®^ Non-Radioactive Cytotoxicity Assay, as previously described [20] or LDH-GloTM Cytotoxicity Assay (J2380 Promega, Madison, WI, USA) with some modifications: combining 10 µL of media from a 96-well plate well with 10 µL of Detection Enzyme and Reductase Substrate, which was premixed just before in the proportion recommended in the protocol and then diluted 1:10 in LDH Storage Buffer (also prepared as recommended in the protocol). To induce the maximum LDH release, 10 µL of 9% (*v*/*v*) Triton^®^ X-100 was added to the wells to permeabilize all cells (100% cell death). Luminescence was measured after 1 h of incubation in the dark in a BioTek Synergy HT Plate Reader using Gen5(TM) software (version 5.02, BioTek Synergy Winooski, VT, USA). Each individual treatment/time point reflects six replicates for all assays performed in RCNs cultured in 96-well plates.

### 4.5. RNA-Interacting Protein Immunoprecipitation (RIP) Using AGO2-Specific Antibodies

One strand of the mature miR binds to Argonaute (Ago) proteins to form the RNA-induced silencing complex (RISC), and the miR acts as a template for the recognition and cleavage of complementary mRNA. miRNA–mRNA target pairs can be purified by immunoprecipitation of the RISC components to confirm mRNA targets.

Ago2 immunoprecipitation was performed as previously described [24] with a few modifications. Briefly, RCNs were suspended in 500 µL of lysis buffer (150 mM KCl, 25 mM Tris-HCl (pH 7.4), 5 mM EDTA, 0.5% IGEPAL CA-630, 5 mM DTT, and RNase Inhibitor (Thermo Fisher Scientific N8080119, Waltham, MA, USA) to a final concentration of 10 U/mL, and protease inhibitor and phosphatase inhibitor (2,3) cocktails (Sigma-Aldrich, St. Louis, MO, USA) at 4 °C for 20 min. Then, the cell lysates were separated by centrifugation at 12,000× *g* for 20 min at 4 °C.

A pre-clearing step was added to reduce non-specific binding. First, 10 uL of Rabbit (DA1E) mAb IgG XP^®^ Isotype Control (# 3900 Cell Signaling Technology, Inc., Danvers, MA, USA) was added to lysates, and the mixture was rotated for 1 h at 4 °C. Then, 50 µL of protein A/G UltraLink Resin (Thermo Fisher Scientific, Waltham, MA, USA) was added to the lysates, and the mixture was rotated for 30 min at 4 °C. After incubation, the beads were removed by centrifugation at 1000× *g* at 4 °C for 5 min. The supernatant was used for immunoprecipitation.

A volume of 50 µL of protein A/G UltraLink Resin (Thermo Fisher Scientific, Waltham, MA, USA) and 20 µL of Argonaute 2 (Ago2) antibody (Cell Signaling, Danvers, MA, USA) were added to 400 µL of cell lysate (in a final 1 mL mixture filled with lysis buffer), and the mixture was rotated for 4 h at 4 °C. The beads were washed three times with 1 mL lysis buffer to remove the nonspecific binding. RNAs bound on the beads were extracted by miRNeasy Kit (Qiagen, Hilden, Germany). miR and gene expression were analyzed by qPCR (as described below). The levels of mRNA and miR-23a-3p were normalized to GAPDH and U6 snRNA (001973 Applied Biosystems, Foster City, CA, USA) levels in inputs, respectively. We controlled the variations in the inputs by normalizing levels of mRNAs to GAPDH and miR-23a-3p to U6 snRNA. We confirmed the specificity of Ago2/RISC IPs by using normal rabbit IgG (#3900 Cell Signaling Technologies, Danvers, MA, USA) for negative control IPs. qPCR analysis confirmed that the levels of target mRNAs and miR-23a-3p in negative control IPs were more than 17 times lower than in IPs with control (nonirradiated) samples.

### 4.6. RNA Isolation

Total RNA was isolated using the miRNeasy Kit (Qiagen, Hilden, Germany). During the process of isolation, samples were treated with RNase-free DNase (Qiagen, Hilden, Germany) to digest DNA contamination of the samples according to the manufacturer’s protocol.

### 4.7. qPCR

A Verso cDNA Kit (Thermo Fisher Scientific, Waltham, MA, USA) was used to synthesize cDNA from purified total RNA as described previously [24]. Water was used instead of RNA in the negative control no-template reaction; water was used instead of an enzyme in the negative control no-enzyme reactions. Quantitative real-time PCR was performed by using a cDNA TaqMan Universal Master Mix II (Applied Biosystems, Foster City, CA, USA). TaqMan Gene Expression assays for the following rat and mouse genes were performed: *GAPDH* (Rn01775763_g1, Mm99999915_g1), *p21* (Rn01427989_s1), *BCL2 binding component 3 (PUMA)* (Rn00597992_m1, Mm00519268_m1), *phorbol-12-myristate-13-acetate-induced protein 1 (Pmaip1 or Noxa)* (Rn01494552_m1, Mm00451763_m1), *Bim* (Rn00674175_m1), *BCL2 associated X, apoptosis regulator (Bax)* (Rn02532082_g1, Mm00432051_m1), *BCL2-antagonist/killer 1 (Bak1)* (Rn01429084_m1), *BH3 interacting domain death agonist (Bid)* (Rn01459517_m1), *BCL2 family apoptosis regulator (Bok)* (Rn01427635_m1), *Bak1*(Rn01429084_m1), *B-cell lymphoma-extra-large (BcL-XL)* (Rn00437783_m1), *B cell leukemia/lymphoma 2 (Bcl-2)* (Rn99999125_m1), *glial fibrillary acidic protein (GFAP) (Mm01253033_m1)*, and *integrin alpha M (Itgam, CD11b)* (Applied Biosystems, Foster City, CA, USA). Reactions were performed in duplicates. Water was used instead of cDNA in the negative no-template control reactions. No-template and no-enzyme negative controls from the reverse transcription step were used to eliminate false-positive results. Reactions were amplified and quantified using a Quant Studio 5 system and the corresponding software (Applied Biosystems, Foster City, CA, USA). Gene expression was normalized to GAPDH, and the relative quantity of mRNAs was calculated based on the comparative Ct method [25].

### 4.8. miR Reverse Transcription and qPCR

Quantitative real-time PCR was used to measure the expression of mature miR-23a-3p. A unit of 10 ng of total RNA was reverse transcribed using a TaqMan miRNA Reverse Transcription Kit (Applied Biosystems, Foster City, CA, USA) with miRNA-specific primers. Water was used instead of RNA in the negative control reaction. Reverse transcription reaction products (1.5 µL) were used for qPCR as described above. TaqMan Gene Expression assays for the following miRs were used: rno-miR-23a-3p (000399, specific for both rat and mouse miR-23a-3p), rno-miR-27a-3p (000408) and U6 snRNA (001973) (Applied Biosystems, Foster City, CA, USA). miRs levels were normalized to U6 snRNA (001973 specific for both rat and mouse).

### 4.9. Isolation of Neurons from the Mouse Brain and qPCR

Part of the cortex and the entire hippocampus were separately dissected from the brain. The parietal regions of the left and right cortices as well as the entire left and right hippocampi were used for RNA isolation. The remaining cerebral hemispheres were used for neuronal isolation by MACS separation technology (Miltenyi Biotec, Bergisch Gladbach, Germany) according to the manufacturer’s instructions. Briefly, brain tissues were rapidly microdissected, and a single cell suspension was prepared using enzymatic digestion (Adult Brain Dissociation Kit; Miltenyi Biotec, Bergisch Gladbach, Germany) in combination with a gentle MACS™ Octo Dissociator. The cells were incubated with Non-Neuronal Cells Biotin-Antibody Cocktail (Miltenyi Biotec, Bergisch Gladbach, Germany) and loaded onto lymphoid cells (LS)columns (Miltenyi Biotec, Bergisch Gladbach, Germany) placed in the magnetic field of a MACS separator. The negative fraction (flow-through neurons) was collected, and the column was washed three times with D-PBS/BSA buffer (Miltenyi Biotech, Bergisch Gladbach, Germany). Non-neuronal positive cells were eluted by removing the magnetic field. The purity of neuronal cell isolation was confirmed by qPCR with pooled neuronal and non-neuronal samples from each group being probed with primers specific to neuronal astrocyte marker GFAP and microglia marker CD11b. The average of levels of GFAP and CD11b mRNAs were approximately 25 times and 100 times lower respectively in isolated neuronal cells compared to non-neuronal cells from the same isolation, respectively (data not shown).

Total RNA was isolated using the Direct-zol RNA Microprep kit (Zymo Research, Irvine, CA, USA) according to the manufacturer’s protocol. First, 200 ng of total RNA was used for cDNA syntheses, as described above. Gene-specific pre-amplification was used to enhance the amount of input material for qPCR. An aliquot of each cDNA sample equivalent to 20 ng RNA was used for pre-amplification with TaqMan Universal Master Mix II (Applied Biosystems, Foster City, CA, USA). The total volume of pre-amplification was 20 μL for each sample. The reaction contained 5 μL of the master mix, 2 μL of cDNA, 5 μL of pooled TaqMan Gene Expression assays, and 8 μL of water. The following temperature protocol was used: 50 °C for 2 min, 95 °C for 10 min, followed by 10 cycles, respectively at 95 °C for 15 s and 60 °C for 1 min. Water was used instead of cDNA in the negative control reaction. The pre-amplified cDNA was 10 times diluted. Then, 2 μL of diluted per-amplified cDNA was used for each qPCR reaction. qPCR was performed as described above.

### 4.10. Cell Lysates Preparation and Western Blot

Whole-cell extracts were prepared, and Western blot was performed as previously described [26]. Chemiluminescence was captured on a ChemiDoc Touch Imaging System (Bio-Rad, Hercules, CA, USA), and protein bands were quantified by densitometric analysis using Image Lab Imaging Software (Bio-Rad). The data presented reflected the intensity of the target protein band compared with the control and were normalized based on the intensity of the endogenous control for each sample (expressed in arbitrary units).

### 4.11. Antibodies

The following antibodies were used in this study: Histone H2A.X (ab11175; Abcam, Cambridge, United Kingdom); cytochrome c (sc-13560; Santa Cruz Biotechnology, Dallas, TX, USA); γ-H2A.X (#9718), phosphorylated Rad3-related serine/threonine kinase (Phospho-ATR) (Ser428) (#2853), Cleaved Caspase-3 (#9661), PARP (#9542), Apaf-1 (#8723), phospho-p53 (Ser15) (#9284), PUMA (#14570), p53 (#2524), Phospho-Akt (Ser473) (#4060; Cell Signaling Technology, Inc., Danvers, MA, USA; GAPDH (ADI-CSA-335) and α-fodrin (BML-FG6090; Enzo Life Sciences, Inc., Farmingdale, NY, USA); β-actin (A1978; Sigma-Aldrich, St. Louis, MO, USA); phosphorylated ataxia telangiectasia (phospho-ATM) (05–740 Millipore, Burlington, MA, USA).

We observed two bands in immunoblots with antibodies against GAPDH. The size of the top band was approximately 37 kDa, which matches the size of GAPDH according to the supplier. Cytochrome c levels were normalized to the top approximately 37 kDa band of GAPDH. The double bands of GAPDH in the cytosolic fraction have been previously reported [27,28].

We observed three bands in immunoblots with antibodies against phospho-ATR (Ser428) for all samples and conditions. The size of the middle band was approximately 300 kDa. According to the supplier, the 300 kDa band is specific for ATR phosphorylation, and this band was used for our analysis.

We observed two bands in immunoblots with antibodies against phospho-ATM (Ser1981) for all samples and conditions. The supplier of Ph-ATR antibodies informed us that in addition to the specific 370 kDa band, these antibodies also detect a nonspecific band >400 kDa. We observed two bands in immunoblots with antibodies against phospho-ATM (Ser1981), for all samples and conditions. The size of the lower band was approximately 370 kDa, which matches the reported size of phospho-ATM, and the intensity of the 370 kDa band was analyzed in our studies.

### 4.12. Subcellular Fractionation

Subcellular fractionation was performed as described previously [24]. RCNs were harvested and washed in ice-cold phosphate-buffered saline. The cell suspension was centrifuged at 500× *g* for 15 min at 4 °C. The cell pellet was resuspended for 10 min on ice in digitonin lysis buffer (20 mM 4-(2-hydroxyethyl)-1-piperazineethanesulfonic acid (HEPES), pH 7.4, 80 mM KCl, 1 mM Ethylenediaminetetraacetic acid (EDTA), 1 mM ethylene glycol-bis(β-aminoethyl ether)-N,N,N′,N′-tetraacetic acid (EGTA), 1 mM Dithiothreitol (DTT), 250 mM sucrose, 200 μg/mL digitonin, protease inhibitor, and phosphatase inhibitor (2, 3) cocktails (Sigma-Aldrich, St. Louis, MO). Cells were passaged 20 times through a 22G needle. The lysate was centrifuged at 1000× *g* for 5 min at 4 °C to pellet the nuclei. The supernatant was transferred to a new tube and centrifuged again at 12,000× *g* for 10 min at 4 °C to pellet the mitochondria. The resulting supernatant, representing the cytosolic fraction, was recovered. Nuclear and mitochondrial lysates were prepared in Radioimmunoprecipitation assay (RIPA) buffer (Teknova) with Protease Inhibitor Cocktail (Sigma-Aldrich, St. Louis, MO). All steps were performed on ice. β-actin beta-actin is a cytoskeleton protein, and that is why it was used to normalize Western blot data that examined from total lysates. GAPDH is a cytoplasm marker, and that is why it was used to normalize Western blot data that were examined from cytosolic fraction after subcellular fractionization.

### 4.13. Immunocytoochemistry

We transfected primary cortical neurons with miR-23a-3p mimic or negative control (non-targeting) mimic 1 h before IR (8 Gy) on DIV 7 in 24-well plates with coverslips. After 30 min, 6 h, or 24 h, RCNs on coverslips were fixed for 10 min in 4% paraformaldehyde/Phosphate-buffered saline (PBS) and then co-stained with a 1:200 dilution of Cell Signaling’s γ-H2A.X (CST #9718, Danvers, MA, USA) antibody and a 1:400 dilution of Millipore’s Milli-Mark™ Pan Neuronal Marker (Burlington, MA, USA) (data not shown) in 10% goat serum (Gemini Bio-Products, West Sacramento, CA, USA) overnight at 4 °C. Wells were incubated the next day with goat-derived secondary antibody (Life Technologies, Fisher Scientific, Hampton, NH), followed by 4′,6-diamidino-2-phenylindole (DAPI, Sigma-Aldrich, St. Louis, MO,) (0.5 µg/mL in saline). Imaging was performed using an Orca^®^-Flash4.0LT Digital CMOS camera (Hamamatsu Photonics K.K., Hamamatsu City, Shizuoka Pref., Japan) mounted on a Nikon Eclipse Ni-E microscope (Nikon, Minato City, Tokyo, Japan) with a Nikon Plan Apo 60X/1.40 OIL WD objective (Nikon, Minato City, Tokyo, Japan). Exposure times and laser power settings were optimized to maximize signal intensity in controls without oversaturating the signal in higher-intensity samples and maintained constant for all images. The analysis was done using Nikon’s NIS-Elements software (version 5.11.01Nikon, Minato City, Tokyo, Japan) and the “General Analysis” tool. Eight separate/non-overlapping fields were selected per coverslip and a series of 2048 × 2044 pixel images was acquired at a resolution of 16 bits with a z-distance of 0.3 µm. Then, each set of images for a given field was used to generate a single image via maximum intensity projection. These maximum intensity projections were analyzed with settings as follows.

DAPI was used to identify nuclei via: Rolling Ball Correction (radius 8.02 µm)-Local Contrast (Size 35, Power 95%)-Threshold-Smooth (2×), Fill holes, Separate (2×)-Object Area filtering (5–120 µm^2^), Morpho Separate Objects (2×). Foci were then identified via γ-H2A.X staining by: Rolling Ball Correction (radius 1.52 µm)-Bright Spot Detection (Typical Diameter = 0.399 µm, Contrast = 713.6).

Analysis was performed on all nuclei containing foci thus identified, and the number of foci or sum of signal intensity of γ-H2A.X within each nucleus was quantified and plotted for all fields together as an unbinned cumulative frequency distribution [29].

### 4.14. Statistical Analysis

All statistics were performed using Graphpad Prism (version 7, Graphpad, San Diego, CA, USA). One-way ANOVAs with Tukey post-hoc tests were used to analyze Western blot, qPCR, ChIP, and LDH assays, except for immunocytochemistry data and cases when only two groups were compared. In those cases, we used a one-tailed *t*-test, while immunocytochemically stained cells were analyzed using the Kruskal–Wallis test followed by Dunn’s post-hoc analysis.

For in vitro LDH, calcein, qPCR, and Western blot assays, at least three separate wells of primary RCN seeded on day 0 from the same primary culture were used for any given assay. These separately cultured and treated neurons isolated from the same pool of embryos were run on the same gel/assay plate and quantified as indicated elsewhere. Each set of experiments was repeated at least twice in an equivalent manner with another pool of embryos from a different pregnant dam and showed consistent results.

## 5. Conclusions

In conclusion, our results suggest that the decline in miR-23a-3p levels after irradiation may contribute to neuronal cell death via the mitochondrial pathway of intrinsic apoptosis [57]. Importantly, the reversal of miR-23a-3p changes by the administration of a miR-23a-3p mimic may attenuate apoptosis not only through a targeted reduction in pro-apoptotic BH3-only PUMA and Noxa mRNA but also by a reduction of upstream secondary DNA-damage with the attenuation of p53 transcriptional activation of apoptosis. The ability of miR-23a-3p to target multiple levels of a key pro-apoptotic pathway may serve to amplify its biological effects. Thus, miR-23a-3p may be part of effective *neuroprotective* therapeutic interventions that may stop the progression of neurodegeneration and promote neuronal survival after IR.
